# Gaze Following in Children with Autism: Do High Interest Objects Boost Performance?

**DOI:** 10.1007/s10803-016-2955-6

**Published:** 2016-12-16

**Authors:** Emilia Thorup, Johan Lundin Kleberg, Terje Falck-Ytter

**Affiliations:** 1 Uppsala Child and Baby Lab, Department of Psychology, Uppsala University, 751 42 Uppsala, Sweden; 2 Karolinska Institute Center of Neurodevelopmental Disorders (KIND), Pediatric Neuropsychiatry Unit, Department of Women’s and Children’s Health, Karolinska Institutet, Gävlegatan 22, 11330 Stockholm, Sweden; 3 Child and Adolescent Psychiatry, Center for Psychiatry Research, Stockholm County Council, Stockholm, Sweden

**Keywords:** Gaze following, Joint attention, Circumscribed interests, Communication, Social cognition

## Abstract

This study tested whether including objects perceived as highly interesting by children with autism during a gaze following task would result in increased first fixation durations on the target objects. It has previously been found that autistic children differentiate less between an object another person attends to and unattended objects in terms of this measure. Less differentiation between attended and unattended objects in ASD as compared to control children was found in a baseline condition, but not in the high interest condition. However, typically developing children differentiated less between attended and unattended objects in the high interest condition than in the baseline condition, possibly reflecting reduced influence of gaze cues on object processing when objects themselves are highly interesting.

## Introduction

We are constantly surrounded by multiple stimuli competing for our attention. When interacting socially, other people’s gaze can constitute important cues as to what stimuli are currently important to attend to. Acknowledging what our interaction partner is looking at often provides information as to what is going on in that person’s head, and thus facilitates interaction as well as social learning. The ability to follow other people’s gaze therefore serves an important function. Gaze following is a crucial aspect of joint attention—the sharing of attention between two individuals and an object (Mundy et al. 1986). Typically developing infants start to engage in joint attention during their first year of life (Corkum and Moore 1998; Gredebäck et al. 2008), and the ability to do so is considered a developmental milestone, critical for later development of communication and social cognition. Decreased engagement in joint attention is one of the most commonly reported characteristics of autism spectrum disorders (ASD; Charman 2003), and an early deviance on this area has been proposed as a common factor behind the later manifest socio-communicative impairments that characterize ASD (Mundy et al. 2009; Tomasello et al. 2005).

Previous research on gaze following in children with ASD has yielded mixed results, with some studies indicating impairment (e.g. Chawarska et al. 2003; Leekam et al. 2000) and others finding typical performance (e.g. Leekam et al. 1998). In a recent eye tracking study (Falck-Ytter et al. 2015), we showed that a group of low-functioning 3-years-old with ASD was equally likely to follow a model’s gaze as typically developing (TD) and developmentally delayed control children. However, when we explored an aspect of the microstructure of the children’s gaze behavior, namely the length of their first fixations to the target objects, a group difference was discovered. For those trials where the children followed the model’s gaze to the attended object before looking at the unattended object, we compared the lengths of the first fixations at each object. The analysis revealed that the children in the two control groups differentiated more between attended and unattended objects in terms of the first fixation durations than autistic children. We suggested that this finding may reflect that another person’s gaze renders an object more interesting or salient for TD children, and changes the interest level or saliency of an object for children with ASD to a lesser extent (Falck-Ytter et al. 2015). A similar finding was made by Swanson and Siller (2013), who found longer first fixations to attended than unattended objects in TD children, but no difference between conditions in children with ASD. Although Swanson and Siller (2013) did not rule out that a group difference in gaze following accuracy could have affected the results, their study and our previous study (Falck-Ytter et al. 2015) both point toward less differentiation between attended and unattended objects in children with ASD compared to typically developing children. The results from these two studies are also broadly in line with a previous study by Bedford et al. (2012) who showed that 13 months old children who were later displaying developmental problems, including ASD, spent less total time looking at the attended object than did typically developing children, but that gaze following accuracy did not predict outcome.

Measures of first fixation duration are not as commonly used in autism research as for example measures of total looking time. Nevertheless, if one is interested in studying processing biases occurring on short timescales and reducing the potential influence of confounding variables such as sustained attention, first fixation duration measures have obvious advantages as they capture differences that occur during the initial fixation (typically <500 ms) linked to an event of interest. In reading research, first fixation duration is believed to reflect the initial lexical activation process (Rayner 1998), and it has been shown that more complicated texts, in terms of both content and grammatical structure, evoke longer fixations. In terms of scene viewing, the measure has been linked to the cognitive processing of the elements in the scene and to visual information acquisition (Holmqvist et al. 2011). For example, it has been shown that first fixations in response to unexpected object combinations are longer than first fixations in response to expected combinations (De Graef et al. 1990; Loftus and Mackworth 1978). Holmqvist et al. (2011) conclude that longer fixations typically are considered an indication of deeper processing.

Preliminary evidence suggests that the first fixation duration measure can reveal the extent to which an individual takes contextual cues such as another’s gaze into account during object processing, and that the measure can discriminate between children with ASD and controls (Falck-Ytter et al. 2015). Currently, with regards to the latter group difference, it is not known whether it applies to all object types, or whether it is restricted to certain object categories. From both a practical and a theoretical perspective, it would be important to know if performance in ASD is improved if certain object types are used.

The aim of the present study was two-fold. First, we wanted to replicate the previous finding of decreased differentiation between attended and unattended objects in the ASD as compared to TD group (Falck-Ytter et al. 2015) in a new sample of older and more high functioning children. Second, we aimed to test whether using objects that are common as circumscribed interests as targets in a gaze following task could improve sensitivity to contextual gaze cues in ASD. Circumscribed interests are highly restricted interests characterized by an abnormal intensity or focus (DSM-5), that have been reported to occur in 75–95% of children with high functioning ASD (Klin et al. 2007; Turner-Brown et al. 2011). Eye tracking studies have revealed that children with ASD show greater visual attention toward objects related to circumscribed interests than TD children (Sasson et al. 2008, 2011), and neuroimaging has shown that viewing such objects results in higher activation of the ventromedial prefrontal cortex—an area associated with reward processing—in individuals with ASD as compared to TD-individuals (Dichter et al. 2012). It can thus be expected that including objects associated with circumscribed interests would increase the general interest level and engagement of autistic participants. Furthermore, intervention studies have shown that objects and games related to circumscribed interests can be used to increase social interaction in general (Boyd et al. 2007; Baker et al. 1998; Baker 2000), as well as joint attention behaviors specifically (Kryzak et al. 2013; Kryzak and Jones 2014). These intervention studies however did not include any looking time measures, and a goal of the current study was to explore whether using objects related to circumscribed interests as gaze targets could lead to increased first fixation durations in ASD. It has previously been demonstrated that looking patterns and performance in a word learning task can be normalized in autistic children when the saliency of the target objects is increased (Akechi et al. 2011) and we reasoned that objects related to circumscribed interests should be particularly salient to the ASD group. We thus predicted that the children in this group would show greater attended-unattended differentiation in terms of first fixation duration when targets belonged to circumscribed interest categories compared to when they did not belong to such categories.

## Methods

### Participants

The final sample consisted of 33 children between the ages of 38 and 112 months (M = 77.73, SD = 15.69; for participant characteristics, see Table 1). The ASD group consisted of 16 children (4 girls), and the TD group of 17 children (5 girls). All children had a non-verbal IQ in the average or above average range, and no children had any uncorrected hearing or visual impairments. Data was collected from an additional 15 children (7 ASD, 8 TD) but excluded from the analysis due to not contributing enough valid data (see “Data Reduction and Analysis” section for details). The children in the ASD group were recruited from the Autism Centre for Young Children in Stockholm, Sweden. All had a community diagnosis of ASD, including Autistic Disorder, Asperger’s Syndrome or Pervasive Developmental Disorder Not Otherwise Specified. The diagnosis was corroborated using the Autism Diagnostic Observation Schedule-2 (ADOS-2; Lord et al. 2012). The TD group was recruited from a database of children whose parents had expressed interest in having their children participate in research on children’s development. Children in the TD group did not have a diagnosis of any medical or developmental condition, including ASD and ADHD, according to parental report. Non-verbal IQ (NVIQ) was determined using the standard non-verbal subtests of either the WPPSI-III (22 children) or, in those cases where the children were too old for the norms, the WISC-IV (8 children). Due to child behavior, NVIQ could not be established for three children in the ASD group (none of whom had a diagnosis of intellectual disability). Autistic traits were assessed in all children using the Social Responsiveness Scale (SRS; Constantino and Gruber 2005). All children in the TD group had total SRS T-scores <60, indicating no clinically meaningful symptoms of ASD. All children in the ASD group but one had total SRS T-scores >60. The remaining child, who had a T-score of 59, was diagnosed with Autistic disorder and had an ADOS-2 score of 12. This child was considered representative of the ASD group and was thus retained in the sample. The Repetitive Behavior Scale-Revised (RBS-R) five-factor solution (Lam and Aman 2007) was used to assess the presence of repetitive behaviors and restricted interests. The ASD group had a significantly higher total RBS-R score than the TD group. Of specific relevance to the current study is the Restricted Interests subscale, where the ASD group also scored higher than the TD group, suggesting a higher presence of circumscribed interests in the former group.

**Table 1:** Participant characteristics by group, final samples (M/SD)

	ASD N = 16 (4 girls)	TD N = 17 (5 girls)	Pairwise comparison (*p* value)
Age (months)	81.56/15.42	74.12/15.51	0.177^a^
NVIQ^b^	105.23/19.20^c^	115.76/11.01	0.068^a^
SRS total score	73.06/25.10	43.65/5.31	<0.001^a,d^
RBS-R total score	20.31/15.78^c^	3.24/3.36	0.002^a,d^
RBS-R restricted interests subscale score	2.85/2.15^c^	0.35/0.61	0.001^a,d^
ADOS-2 total score	14.31/5.77	N/A	N/A

^a^Independent samples *t*-test

^b^NVIQ was assessed using the standard non-verbal subtests of WPPSI-III or WISC-IV

^c^Based on 13 children

^d^Adjusted due to unequal variances

All parents provided written informed consent. The study was approved by the Regional Ethical Board in Stockholm, and conducted in accordance with the standards specified in the 1964 declaration of Helsinki.

### Procedure

The eye tracking experiment was administered in the beginning of the visit. The child was seated at a distance of 60 cm in front of a 17-inch screen (Tobii T120, Danderyd, Sweden), where the stimuli were presented. A 5-point calibration was conducted before the start of the stimuli presentation, and repeated if needed. The stimulus videos were intermixed with other stimuli and attention grabbers and presented in three blocks, with short breaks in between. The order of the videos was pseudo-randomized so that no gaze following clips occurred directly after each other, and no more than two clips from the same condition occurred in a row. The WPPSI-III/WISC-IV and the ADOS-2 (ASD group only) were administered at the same visit as the eye tracking. The SRS and RBS-R were completed by the parents before the visit.

### Stimuli

**Fig. 1:**
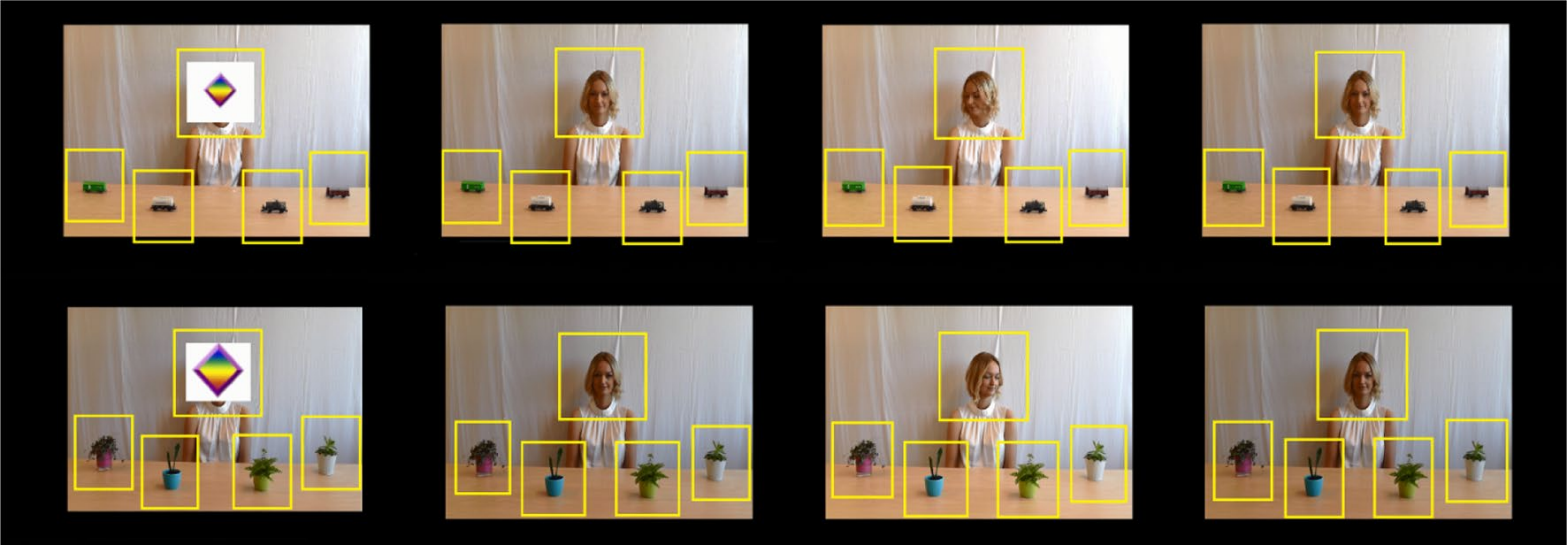
Screen shots of the stimulus video, showing the attention grabber covering the model’s face, the model engaging in direct gaze, the model attending to one of the objects and then engaging in direct gaze again. Areas of interest (AOIs) are highlighted. Upper row depicts one of two stimuli sets in the high interest condition; lower row depicts one of two stimuli sets in the baseline condition

The stimuli were 16 videos (duration 10 s each; see Fig. 1) of a female model seated behind a table, with four objects in front of her. We increased the number of target objects from our previous study of younger low functioning children with ASD (Falck-Ytter et al. 2015) in order to better match the current sample, that was older and had higher IQ. For the first 1.5 s. of the video, the model’s face was covered by an animated attention grabber. A female voice said “hello!” and the attention grabber then disappeared, revealing the model looking straight ahead, smiling slightly. The model then started turning her head toward one of the four objects. The duration of each head turn was 0.5 s. The model kept looking at the object for another 5 s, before turning her head back (0.5 s.) and looking straight ahead for the remaining 2.5 s. The high interest condition included two sets of objects, one with model trains and one with toy vehicles. Both trains and vehicles are documented common interest areas in ASD, and have previously been used in studies measuring attention to objects related to circumscribed interests (Sasson et al. 2008, 2011; Sasson and Touchstone 2013). The baseline condition also included two sets of objects, one with flowering house plants and one with house plants with green leaves. The plants were roughly the same size as the trains/vehicles, and we aimed for the different object types to be somewhat equivalent in terms of perceptual detail. Plants were chosen to represent ordinary objects, regularly seen by most children and uncommon as circumscribed interests. Eight videos included trains/vehicles and eight videos included plants. Each of the four objects comprising a stimulus set was attended to once by the model.

### Data Reduction and Analysis

Gaze data was recorded at a sample rate of 60 Hz, and analyzed using the Tobii Studio 3.2.3 software (Tobii Technology, Stockholm, Sweden). In accordance with our previous study (Falck-Ytter et al. 2015), a fixation filter (Tobii Fixation Filter) with a velocity threshold of 35 pixels/window and a distance threshold of 35 pixels was applied. All fixations shorter than 60 ms were discarded. Seven rectangular areas of interest (AOIs) were defined. One covered the attention grabber in the beginning of the video. The remaining six were activated after the removal of the attention grabber and covered the face of the model, the four objects as well as the total screen area. The face and attention grabber AOIs subtended 8.9 by 8.4 visual degrees, and the object AOIs subtended 5.8 by 6.5 visual degrees.

First fixation durations within each AOI were extracted from when the model’s face was revealed until the end of the clip. Gaze shifts were coded manually, using gaze replays (recordings of the stimuli with the participant’s gaze and AOIs superimposed). *Congruent* gaze shifts were coded whenever a fixation was first recorded in the face AOI and subsequently in the AOI covering the attended object, without first entering the AOIs on the unattended side. *Incongruent* gaze shifts were coded whenever a fixation was first recorded in the face AOI and subsequently in any of the two object AOIs on the unattended side, without first entering the AOI covering the attended object. The visual inspection of the videos revealed that it was relatively common for children of both groups to start looking toward objects immediately after the model started her gaze shift, relying on the general direction of her gaze rather than paying attention to which specific object she attended to. This resulted in a substantial number of looks to the unattended object at the same side as the attended object. Due to the difficulty of teasing apart whether those responses reflect “accurate” gaze following based on the general direction of gaze or an “inaccurate” response (see Falck-Ytter 2012), all such trials were removed from the main analyses. The number of these trials did not differ between groups.

Statistical analyses were performed in SPSS (SPSS Inc., Chicago, IL). Three dependent measures were defined as follows: (1) Accuracy was calculated as a proportion where the number of congruent gaze shifts was divided by the total number of (congruent and incongruent) gaze shifts. Because trials where the child looked at the object next to the attended object first were removed from the analyses (see “Data Reduction and Analysis” section) an accuracy score of 0.33 would indicate chance performance, and we expected the performance of each group to exceed this value in both conditions. (2) For the analysis of first fixation durations, we started by calculating the mean first fixation durations at attended and unattended objects in each condition. The averages were then used to calculate a proportional measure where the first fixation duration at attended objects was the nominator and the sum of the first fixation durations at attended and unattended objects was the denominator. We termed this measure the Attended—Unattended Fixation Index (AUF-index). Values above 0.5 would indicate longer first fixations at attended than unattended objects, whereas values below 0.5 would indicate longer first fixations at unattended objects (but see “Discussion” section for why it may be incorrect to consider 0.5 as a value representing no first fixation bias). In accordance with the previous study (Falck-Ytter et al. 2015) and because our focus of interest was the performance on trials when the children *did* follow gaze, only data from trials with congruent gaze shifts was included in this analysis. We chose not to use a difference score as in our previous paper (Falck-Ytter et al. 2015), since this resulted in non-normally distributed data. In order to check how well the AUF-index and the previously used difference score corresponded to each other, the data from the previous study was reanalyzed using an AUF-index. The pattern of statistical results was the same across these two analyses. (3) Total looking time at objects was defined as the total duration of fixations at objects divided by the total duration of fixations at the screen, thus resulting in a measure indicating the proportion of time that was spent looking at objects. All trials, regardless of whether the children followed gaze or not, were included in this analysis, as were looks to attended as well as unattended objects. Since total looking time is a measure known to indicate interest (Holmqvist et al. 2011), the purpose of this measure was to assess object type preferences. We expected the autistic children to spend more time looking at the objects in the high interest as compared to baseline condition, but did not have any specific hypothesis regarding this measure in the TD group. To be included in the analysis, each child had to contribute at least two valid trials per condition to the accuracy analysis, and, given the lower total number of trials, at least one valid trial per condition to the first fixation duration analysis.

## Results

### Gaze Following Accuracy

The gaze following accuracy of both groups was, as expected, greater than chance in both the high interest condition (ASD: *t*(15) = 8.24, *p* < 0.001; TD: *t*(16) = 6.05, *p* < 0.001) and the baseline condition (ASD: *t*(15) = 6.08, *p* < 0.001; TD: t(16) = 5.11, *p* < 0.001; one sample *t*-tests comparing the means to 0.33). A repeated measures ANOVA on the accuracy scores revealed no main effect of condition, *F*(1, 31) = 0.10, *p* = 0.756, partial *η* ^2^ = 0.003, or group, *F*(1, 31) = 2.49, *p* = 0.125, partial *η* ^2^ = 0.07, and no interaction effect between group and condition, *F*(1, 31) = 0.02, *p* = 0.878, partial *η* ^2^ = 0.001. For descriptive statistics, see Table 2.

**Table 2:** Descriptive statistics by group (M/SD) for accuracy measures

Measure	ASD	TD
N valid trials, high interest condition	3.88/0.96	4.47/1.01
N valid trials, baseline condition	4.38/1.31	4.94/1.20
Accuracy^a^, high interest condition	0.75/0.20	0.64/0.21
Accuracy^a^, baseline condition	0.73/0.26	0.63/0.24

^a^N congruent gaze shifts divided by total N (congruent and incongruent) gaze shifts

### First Fixation Durations

**Fig. 2:**
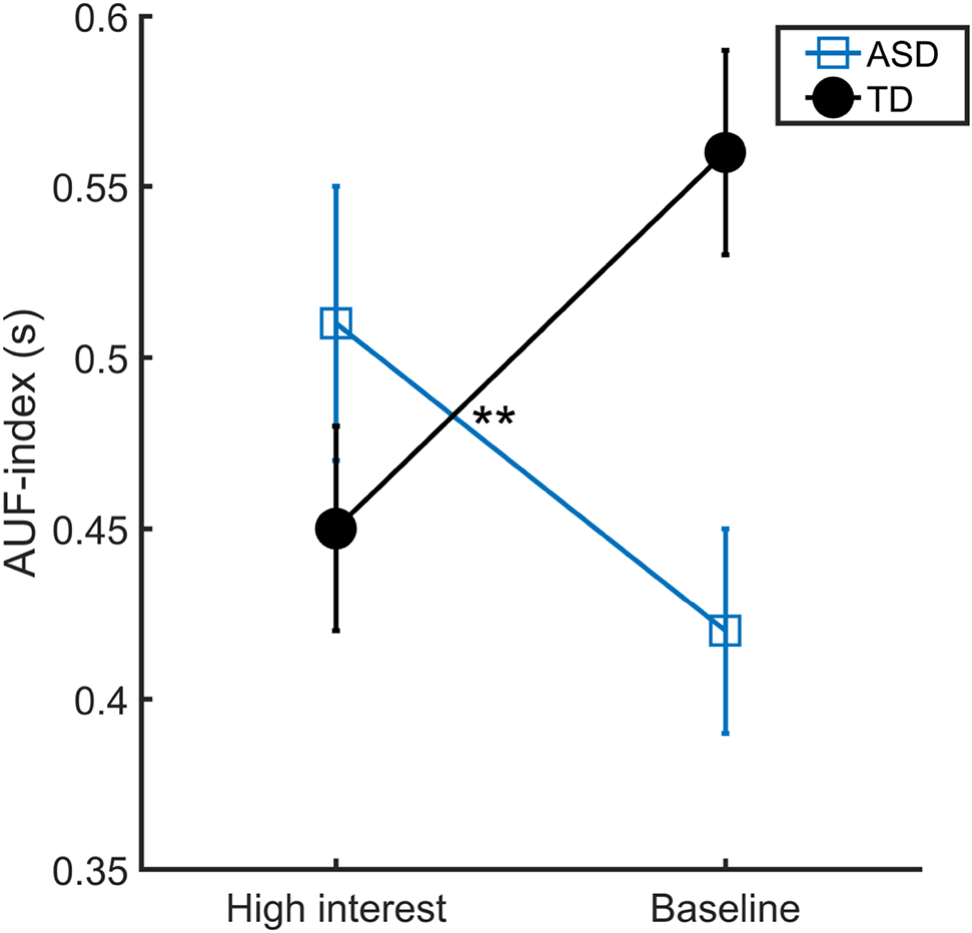
AUF-index (FFD at attended object divided by sum of FFD at attended and unattended objects, averaged across trials) for the two groups across conditions (*error bars* represent standard errors). **p < 0.01

To test our main hypothesis, namely whether the two groups were affected differently by the object manipulation in terms of the AUF-index, a repeated measures ANOVA with condition as within subjects variable and group as between subjects variable was performed on this measure. The analysis revealed a group by condition interaction effect, *F*(1, 31) = 11.31, *p* = 0.002, partial *η* ^2^ = 0.27, but no main effect of condition, *F*(1, 31) = 0.13, *p* = 0.721, partial *η* ^2^ = 0.004, or group, *F*(1, 31) = 0.86, *p* = 0.362, partial *η* ^2^ = 0.03 (see Fig. 2). In order to understand the interaction effect, a series of follow-up tests (Bonferroni corrected for four comparisons) were performed. As expected (Falck-Ytter et al. 2015), a higher AUF-index was found in the TD as compared to ASD group when the children looked at objects that are typically not the focus of circumscribed interests (baseline condition), *t*(31) = 2.95, *p* = 0.024, *d* = 1.06. Moreover, as expected, no difference between groups was found in terms of the AUF-index when the objects belonged to such categories (high interest condition), *t*(31) = 1.47, *p* = 0.608, *d* = 0.53 (independent samples *t*-tests). Contrary to our expectations however, no difference between conditions was found in the ASD group, *t*(15) = 1.65, *p* = 0.476, *d* = 0.85, but in the TD group a significantly higher AUF-index was found in the baseline as compared to high interest condition, *t*(16) = 4.06, *p* = 0.004, *d* = 2.03 (paired samples *t*-tests). For descriptive statistics, see Table 3.

**Table 3:** Descriptive statistics by group (M/SD) for first fixation duration raw scores

Measure type	Specific measure	ASD	TD
N valid trials, high interest condition		2.81/0.83	2.82/1.01
N valid trials, baseline condition		3.13/1.36	3.12/1.36
First fixation duration raw scores (s)	Attended object, high interest condition	0.38/0.27	0.36/0.31
	Unattended objects, high interest condition	0.32/0.13	0.41/0.15
	Attended object, baseline condition	0.30/0.13	0.54/0.39
	Unattended objects, baseline condition	0.44/0.30	0.39/0.23
First fixation duration raw scores at objects irrespective of gaze following performance (s)	High interest condition	0.36/0.08	0.36/0.10
	Baseline condition	0.37/0.11	0.39/0.15

### Total Looking Time at Objects

**Fig. 3:**
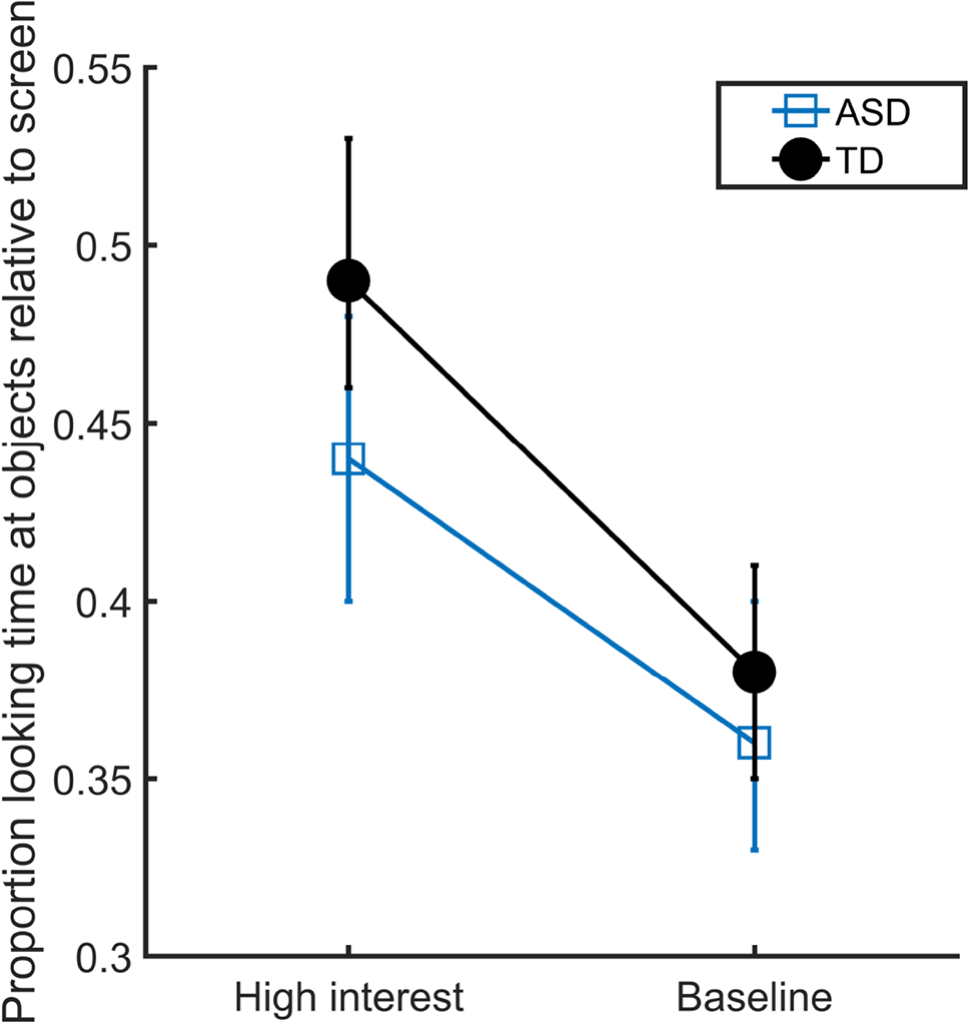
Total looking time at objects divided by total looking time at screen for the two groups across conditions (*error bars* represent standard errors)

A repeated measures ANOVA with total looking time as the dependent measure, condition as within subjects factor and group as between subjects factor revealed a main effect of condition, *F*(1, 31) = 11.77 *p* = 0.002, partial *η* ^2^ = 0.28, with more looking time at high interest compared to baseline objects (see Fig. 3). No main effect of group, *F*(1, 31) = 0.64, *p* = 0.429, partial *η* ^2^ = 0.02, and no interaction effect between group and condition, *F*(1, 31) = 0.38, *p* = 0.540, partial *η* ^2^ = 0.01, was found. The analysis thus showed that the children, regardless of group status, looked more at the objects in the high interest condition than in the baseline condition.

### Supplementary Analyses

The groups did not differ in terms of the number of valid trials in the accuracy analysis in either the high interest condition, *t*(31) = 1.74, *p* = 0.092, *d* = 0.63 or the baseline condition, *t*(31) = 1.30, *p* = 0.204, *d* = 0.47 (independent samples *t*-tests). The number of valid trials in the first fixation duration analysis also did not differ between groups in either the high interest condition, *t*(31) = 0.03, *p* = 0.973, *d* = 0.01 or the baseline condition, *t*(31) = 0.02, *p* = 0.988, *d* < 0.01. For descriptive statistics, see Tables 2 and 3.

To evaluate whether there were general differences in first fixation durations between groups or objects types, a separate analysis was conducted on the mean first fixation duration on all objects and trials, irrespective of gaze following performance. A repeated measures ANOVA revealed no main effects of condition, *F*(31, 1) = 0.57, *p* = 0.455, partial *η* ^2^ = 0.02, or group, *F*(31, 1) = 0.08, *p* = 0.775, partial *η* ^2^ = 0.003, and no interaction between group and condition, *F*(31, 1) = 0.22, *p* = 0.643, partial *η* ^2^ = 0.007.

No correlations between AUF-index and NVIQ were found in either the high interest condition (ASD: *r* = 0.25, *p* = 0.408; TD: *r* = 0.20, *p* = 0.442) or the baseline condition (ASD: *r* = 0.17, *p* = 0.574; TD: *r* = 0.19, *p* = 0.468). Neither were any significant correlations between AUF-index and any ADOS-2, SRS or RBS-R scores found.

Finally, the main AUF-index analysis was rerun with age and non-verbal IQ entered as covariates. The group by condition interaction effect remained significant, *F*(1, 26) = 7.00, *p* = 0.014, partial *η* ^2^ = 0.22, and no interaction effects between condition and age, *F*(1, 26) = 0.01, *p* = 0.942, partial *η* ^2^ < 0.001, or NVIQ, *F*(1, 26) = 0.07, *p* = 0.798, partial *η* ^2^ < 0.01 were found. Note that the N in the ASD-group for this analysis is 13, since it was not possible to establish NVIQ in three of the children.

## Discussion

The aims of the present study were to replicate the group effect observed by Falck-Ytter et al. (2015) in a new sample of older and more high functioning children, as well as to test whether using objects that are common as circumscribed interests as targets in a gaze following task could result in longer first fixations to attended objects in the ASD group compared to objects that are not linked to circumscribed interests. In order to compare the lengths of the first fixations, an Attended-Unattended Fixation Index (AUF-index; see “Data Reduction and Analysis” section) was calculated. As expected, the analysis revealed a lower AUF-index in the ASD as compared to TD group in the baseline condition, where the target objects were plants, chosen to represent ordinary objects that do not commonly represent circumscribed interests in ASD. This is in line with our previous finding (Falck-Ytter et al. 2015) of less differentiation between attended and unattended objects in terms of the first fixation duration in a group of low-functioning 3-year-olds. By extending the finding to older and more high functioning children, the current results strengthen the view that the first fixation duration measure reliably discriminates between TD and ASD groups at different ages and at different levels of intellectual functioning. As in our previous study (Falck-Ytter et al. 2015), both groups followed gaze above chance level and the group difference in the AUF-index could not be explained by lower gaze following accuracy in the ASD group. Notably, in the present study there was even a trend toward *higher* gaze following accuracy in the ASD group than TD group. The present results thus add to the converging evidence showing that in a laboratory based task with a model using both head and eyes as cues, children with or at risk for ASD are as likely to follow gaze spontaneously as typically developing children (Leekam et al. 1998; Bedford et al. 2012; Falck-Ytter et al. 2015; Thorup et al. 2016). This is interesting considering that clinical observations indicate prominent problems with joint attention behaviors in the everyday life of autistic children. Due to methodological issues such observations are not informative regarding the mechanisms behind these problems, which is why experimental studies are needed to complement the clinical observations. That many such experiments fail to demonstrate group differences in terms of gaze following accuracy indicates that the mechanisms behind the real life impairment are more subtle than e.g. a mechanistic inability to follow another’s eye/head movement.

The results showed that children, irrespective of group, spent more time looking at trains and vehicles than at plants, suggesting that we succeeded in manipulating the interest level of the objects. Moreover, the analysis of the AUF-index revealed the expected group by condition interaction effect, showing that the object type manipulation affected the two groups differently. Contrary to our hypothesis however, no significant effect of object type was found in the ASD group, and including interesting objects hence did not affect the performance in terms of first fixation durations. Instead, we discovered that the typically developing children displayed a significantly higher AUF-index in the baseline as compared to high interest condition, demonstrating that they differentiated more between attended and unattended objects when the objects were plants than when they were trains and vehicles. Considering that the children spent more total time looking at the trains and vehicles, the finding is unlikely to reflect that the typically developing children found the plants more interesting than the vehicles. Rather, it seems that in typically developing children, the AUF-index may decrease with increasing interest level of the target objects. Considering that this finding was unexpected, we must be cautious interpreting it. However, it is possible that another’s gaze is taken more into account when the other is looking at a less interesting object, as compared to when the attended object itself is perceived as highly interesting or attention grabbing. In other words, the model’s gaze toward the plants leads to an increase in first fixation duration to attended objects since the plants themselves are not perceived as particularly interesting. The trains/vehicles on the other hand, are perceived as interesting regardless of the gaze cue, which therefore has a weaker effect.

Together, these findings suggest that autistic children do not differ from TD children in terms of gaze following accuracy, but that group differences arise with regards to the subsequent processing of the target objects. It has previously been suggested that children with or at risk for ASD might not perceive gaze cues as communicative to the same extent as other children (Bedford et al. 2012). Using the visual attention of one’s interaction partner as guidance during object processing facilitates interaction and communication—areas where individuals with ASD are known to experience difficulties. An attenuated influence of other people’s gaze cues could well be one of the factors behind these difficulties. The current study further shows that the AUF-index is a measure that captures subtle differences in gaze following behavior that would not be possible to detect with the naked eye. This underlines the unique potential of eye tracking to describe the microstructure of gaze atypicalities in ASD.

Although both the current and previous studies (Falck-Ytter et al. 2015; Swanson and Siller 2013) indicate that others’ gaze cues have an attenuated effect on object processing in ASD as compared to TD, the specificity of this effect is not known. Less modulation of fixation durations in ASD has been reported in other contexts as well. Benson et al. (2012) discovered that adults with ASD differentiated less between contextually congruent (e.g. a car on a highway) than incongruent picture combinations (e.g. an elephant on a highway) than neurotypical individuals in terms of the first fixation duration. Wass et al. (2015) discovered that whereas fixation lengths of typically developing infants increased with time during scene viewing (a pattern that has repeatedly been established in adults, which will be accounted for in the next paragraph) no such time effect was found in a group of infants at high risk for ASD (Wass et al. 2015). Together, these findings might indicate that fixation lengths are less susceptible to contextual factors in general and perhaps less affected by top-down processes in ASD than in typical development. Several accounts have focused on broad differences in perceptual and cognitive functioning between autistic and TD individuals. The “Enhanced Perceptual Functioning” model (Mottron et al. 2006), and the “Predictive Coding” account (Van Boxtel and Lu 2013) both suggest a favoring of local over global processing as a potential core mechanism behind the autistic phenotype. Pellicano and Burr (2012) argue that individuals with ASD are less influenced by previous experiences when processing sensory information, which they attribute to reduced top-down processing (but see Brock 2012 for a comment on how the same phenomenon can be explained by enhanced bottom-up processing). Elsabbagh and Johnson (2016) recently argued that instead of viewing ASD as comprised of primarily social symptoms, early differences should be searched for in widespread neuronal networks underpinning motor, perceptual, attentional and social functioning. Regardless of whether the current results reflect a specific insensitivity to gaze cues in ASD or whether they reflect a more domain general difference, they add to our understanding of the relation between gaze behavior and contextual factors. Even if the more general explanation applies, a lower modulation of gaze behavior could still have specific consequences in the social domain for individuals with ASD. Further research on fixation lengths in both social and non-social tasks is however needed.

Like in our previous study (Falck-Ytter et al. 2015), we decided not to test individual AUF-index scores against a baseline. If fixations were stable over time, an AUF-index of 0.5 would represent no first fixation bias. However, as it has been repeatedly shown that fixation durations increase over time during scene viewing (e.g. Pannasch et al. 2008; Irwin and Zelinsky 2002; Galpin and Underwood 2005; Antes 1974), and as the current design entails that attended objects were always fixated before unattended objects, we would expect a value lower than 0.5 if the model’s gaze had no influence on the first fixation lengths. Interestingly, a recent study of infants at high and low risk for ASD (Wass et al. 2015) found the expected pattern of increasingly longer fixations in the low risk group, but found that fixation lengths in the high risk group did not increase with time. It is important to note, that if the pattern of increasing fixation lengths is indeed absent in ASD but present in TD, it would bias AUF-indexes upward in the ASD group relative to the TD group. The current finding of a lower AUF-index in the ASD group in the baseline condition as well as the previous findings by Falck-Ytter et al. (2015) and Swanson and Siller (2013) can therefore not be explained by this. Wass et al. (2015) also found generally shorter fixations in high risk than low risk infants. This underlines the importance of using a relative measure such as the AUF-index that focuses on the relationship between fixations at attended and unattended objects within each group. It is notable however, that no group difference in first fixation lengths when gaze following accuracy and object status (attended or unattended) was disregarded was found in the current study.

The current study has some limitations. First, even though the groups did not differ significantly in terms of non-verbal IQ, the non-verbal IQ of the control group was above average. No correlations between NVIQ and AUF-index were however observed, suggesting no effects of NVIQ on the results. Second, we did not include a measure of verbal IQ. Considering the non-verbal nature of the task, it seems unlikely that verbal ability should have affected the results, but future studies should nevertheless aim to include such a measure. The small sample sizes and the relatively low number of valid trials must also be considered a limitation. Some data loss was inevitable, considering that the focus of the study was first fixation durations when the children *did* follow gaze, which entails that all trials where gaze following did not occur had to be excluded from the main analysis. Replication in a larger sample with an increased total number of trials is however desired.

Future studies should aim to further explore the relation between gaze cues, first fixation durations and object properties in typical development as well as in ASD. In the current study we measured total fixation durations to the objects in order to validate that the high interest objects were indeed perceived as more attractive than the baseline objects. In the future, one could test this more directly, e.g. by having the participants rank the attractiveness of the objects, or by individualizing the paradigm by using objects preferred by each participant. Whereas the current study aimed to compare objects perceived as interesting vs. neutral, other categories such as objects that are disliked or aversive, or (familiar and unfamiliar) humans could also be included. Greater care should also be taken to control for low level features that may differ between the objects making up the different conditions.

In conclusion, the current results validate the AUF-index as a measure for detecting subtle differences in gaze following behavior (see Falck-Ytter et al. 2015; Swanson and Siller 2013). In addition, our findings indicate that in typically developing children, the effect of other people’s gaze cues on first fixation durations at objects in a scene changes as a function of the interest level of the objects. In the ASD group, no such effect was found. Although more research is needed to determine the relation between gaze cues, first fixation durations and object properties, the present study enhances our understanding of how other people’s gaze affects object processing in ASD as well as in typical development.
